# Supplementary material for: The causal relationship between the human gut microbiota and pyogenic arthritis: a Mendelian randomization study
Source: Front Cell Infect Microbiol. 2024 Nov 26;14:1452480. doi: 10.3389/fcimb.2024.1452480 (PMC11629706; doi:10.3389/fcimb.2024.1452480)
Supplement: Supplementary file 1 [file DataSheet1.zip › mendelian test/ebi-a-GCST90027591.csv_forest.pdf]

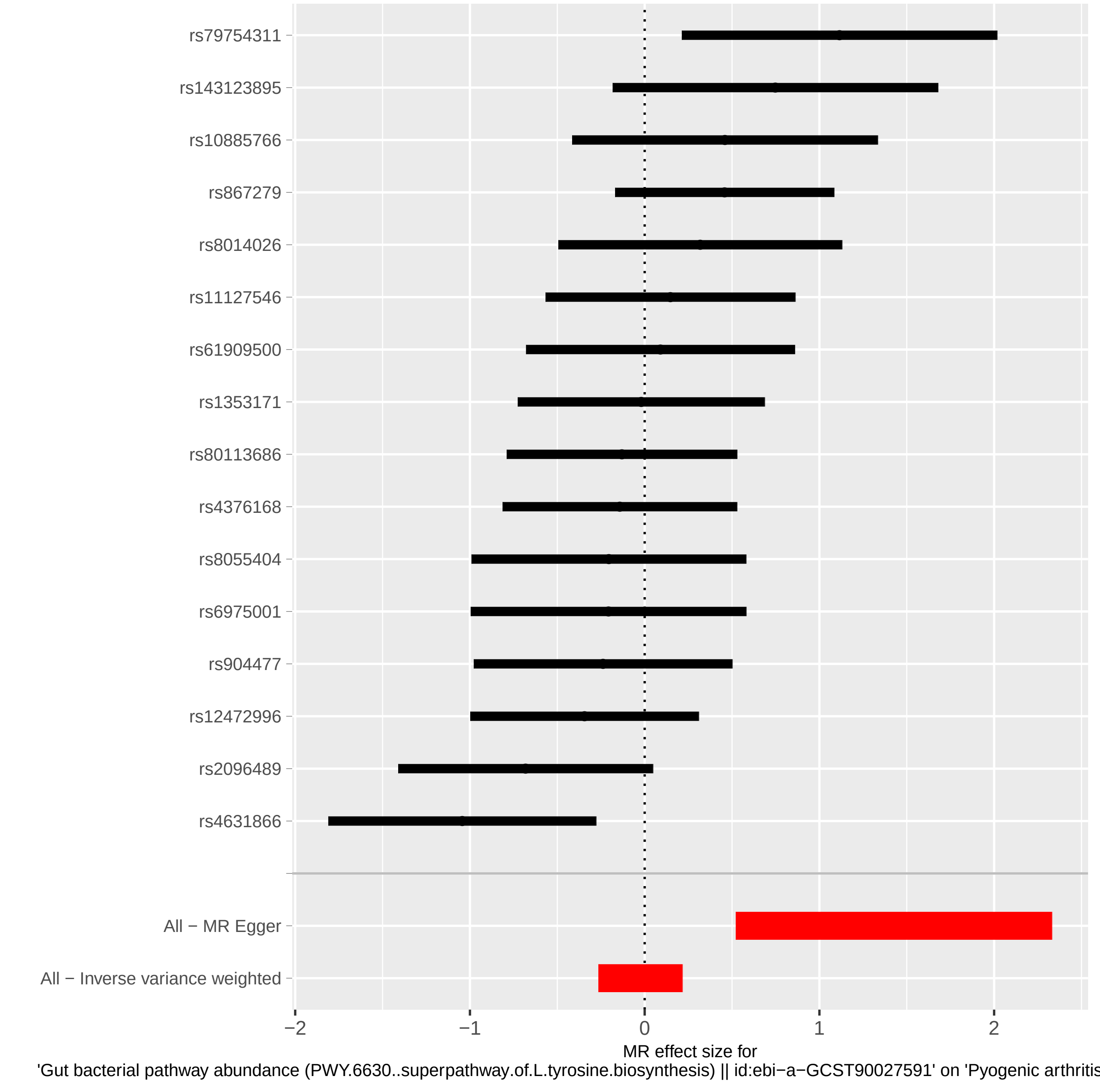
rs79754311
rs143123895
rs10885766
rs867279
rs8014026
rs11127546
rs61909500
rs1353171
rs80113686
rs4376168
rs8055404
rs6975001
rs904477
rs12472996
rs2096489
rs4631866
All − MR Egger
All − Inverse variance weighted
−2
−1
0
1
2
MR effect size for
'Gut bacterial pathway abundance (PWY.6630..superpathway.of.L.tyrosine.biosynthesis) || id:ebi−a−GCST90027591' on 'Pyogenic arthritis
